# On the Effects of Temperature in Pulmonary Consumption

**Published:** 1815-08

**Authors:** Thomas Sutton


THE LONDON

### Medical and Physical Journal.

2 OF VOL. XXXIV.] AUGUST, 1815. [NO. 198.

"For many fortunate discoveries in medicine, and for the detection of numerous errors, the world is indebted to the rapid circulation of Monthly Journals; and there never existed any work to which the faculty in Europe and America were under deeper obligations than to the Medical and Physical Journal of London, now forming a long, but an invaluable, series."—Rush.

*For the Medical and Physical Journal.*

### *On the Effects of Temperature in Pulmonary Consumption;* by Thomas Sutton, M.D.

IN my communication on Pulmonary Consumption, which was published in your Number for March, I endeavoured to show that we had as good grounds for imitating the atmosphere of the colder climates in the cure of this disease, as for attempting it by a high temperature, if we took the infrequency of the disease in different climates, as the standard by which the treatment, as to temperature, should proceed. This was a deduction from facts, acknowledged to be tolerably accurate by parties who favoured the hot treatment. In the same paper also I considered it to be far from proper to infer, because a disease is infrequent in any stated country, when it does actually occur there, that it should of course be mild or more manageable than in a country in which it occurred frequently; and that no just conclusion could be drawn from such information on such a subject; but that the test of this must rest upon experience alone. In the same paper, I stated also that it could not be a necessary consequence, because a certain temperature, such as cold, might contribute much to occasion a disease; that therefore such a disease required to be treated in a warm atmosphere as its opposite, and a low temperature, in all its circumstances, avoided.—In Dr. Buxton's reply to me, in your Number for May, I do not find that he attempts any direct refutation of these statements, although it was very evident that he relied much upon a contrary consideration of them, for the purpose of confirming the propriety of his treatment of consumption by heat, either in the nature of direct evidence or as strong collateral proof of its propriety.

Having therefore, I believe, much weakened his mode of defence, I shall now proceed to consider the material part of his reply, which consists of a sort of evidence, that pulmonary consumption is more successfully treated in hot climates than in this country. For this purpose, I shall very distinctly remark the tendency of the evidence we have on this subject, in so far as it affects the disease actually formed, and in so far as it refers to the incipient state. The first of these is more connected with the institution Dr. Buxton is endeavouring to establish than the latter. The incipient stage of pulmonary consumption is very frequently passed over without great inconvenience. Such, therefore, as gain their livelihood by their daily exertions, frequently unsuspiciously allow the disease to creep on, until they become incompetent to any useful exertion. Before they have arrived at this period, they seek for little aid, but are now willing to adopt any plan for their relief.

At this time they are willing to take the advantage of any institution that offers itself for their recovery, and to consent to a separation from their family and friends, for that purpose. This must also be the time, when they will be willing to consign themselves to the care of Dr. Buxton's institution, and this is also the period in which I have principally contended, that the temperature he has proposed for their cure is, as a general rule, disadvantageous, carries the patient on more rapidly to dissolution, and affords a more limited chance of recovery.

In my letters on consumption, I have given my reasons for forming this opinion, and I have now to examine, whether any evidence we possess, respecting the effects of a warmer climate than our own on the disease, has a tendency to warrant the use of artificial heat for the cure of consumption in this stage of the complaint.

Dr. Gourlay states, " that a change of climate can be then only beneficial when the malady is in its first stage, and the lungs have suffered little derangement in their structure and functions." This is said evidently in reference to observations made on the disease in Madeira. In another part he says, " when the real pulmonary symptoms commence, they prove more violent and rapid in their progress, than in the phthisis of northern climates." And this, although said of the disease in reference to the inhabitants of Madeira, is no-where contradicted respecting its progress on strangers. My private information, indeed, agrees in this respect, that those labouring under the actual and confirmed disease, sink very rapidly in the climate of Madeira.

Dr. Gladstone's evidence also goes to confirm this opinion, in which he joins the other medical men in the naval department, who had arrived at a rule, to send their consumptive patients from the Mediterranean to a northern climate, as the only chance of their recovery. Dr. Domeier, also, about whose account of the climate of Malta much is said by Dr. Buxton, is only " inclined to think, (not from his own experience to assert,) that the climate would contribute much to the cure of consumption at an early period of the disease."

Dr. Lempriere also, who has favoured me with a private communication respecting the disease in Jamaica, says, " patients, when in the incipient state, or rather when predisposed to the disorder (consumption) in this country, may with reasonable hopes be sent to the West Indies. To this I beg to add, if the measure be deferred beyond that period, I have uniformly observed that they sink very rapidly."

In a private letter from Dr. Burnett to Dr. Gladstone, the former says, " With respect to consumptive complaints receiving benefit from a residence in the Mediterranean, I can only say, that, out of many hundreds, I never saw one, during any season of the year, whose case was in any shape ameliorated by it. The summer is decidedly of injury, and, in that season, consumptive complaints advance with great rapidity (in general) to a fatal termination. I know several instances, wherein a removal to a northern climate has been of much service."

I ought here to remark, that Dr. Burnett was for a considerable time at the head of the naval medical department in the Mediterranean. Dr. Irvine is the only author who speaks very positively respecting the benefits of a hot climate in the confirmed stage of consumption, in his account of the disease, as it existed in Sicily, under his observation. He says, " Under this management, I have seen the progress of many cases of Phthisis arrested; and many men recover, for a time at least, a perfect state of health, who would, in all probability, have died in England."

I leave it to every one to draw his own conclusions, why these men perfectly recovered, only, for a time at least. In England, although the disease is considered as very fatal, we see patients recover from an apparent state of confirmed consumption to a perfect re-establishment of health, although these cases are not so frequent as could be wished.

But let us hear from the same author what the feelings of the natives of Sicily are respecting the disease. " Not only does its fatality excite their terror, but an universal persuasion of its infectious nature renders the unhappy patient an object of horror to his relations." With the Sicilians themselves, therefore, the impressions respecting the fatality of the disease, are full as strong as in this country.

The above authorities cannot, I judge, fail to lead to an inference in every mind, that the climate of hot countries is inimical to the recovery of patients in any other than the very first stage of consumption; and bring into great doubt the propriety of confining the consumptive in a factitious high temperature, in this country, for the cure of the disease.

Dr. Buxton has taken the trouble of preferring inference to positive testimony, on one part of the subject under discussion. He thinks, because several authors, who have written on the diseases of the West-Indies, have not mentioned consumption in any direct terms, that therefore it hardly exists there; although Dr. Lempriere has given a table of deaths by the disease as it occurred in Jamaica. In like manner he places Cleghorn as the author to be relied on for the infrequency of the disease in the Mediterranean, although Dr. Irvine speaks of it as very common in Sicily, and he takes the testimony of Domeier as very important in regard to Malta, who appears hardly to have thought of the disease. But, even admitting that it was very unfrequent in the warm climates, he cannot possibly ground any argument upon this, unless he can prove when consumption occurs, that its fatality is in much less proportion and slower in its progress than in this country; the contrary to which I have manifestly shewn to be the case, in so far as respects the actually formed disease. Dr. Buxton is, however, very anxious to impress his readers with the opinion, that this country is pre-eminent in its sufferings respecting consumption, and that it is a disease of very much more frequent occurrence than either in Madeira or in Sicily, because neither Dr. Irvine or Gourlay speak of this complaint in so prominent a manner as he thinks a foreigner would of consumption in speaking of the diseases of England. These authors, however, state, that it prevails in the respective countries alluded to, in very open terms.

Dr. Gourlay says, "No malady is more prevalent than Phthisis with the natives of this island:" and Dr. Irvine says, "It is an ordinary and dreaded disease among them." I have also something to add to our information respecting the occurrence of consumption in the West-Indies, from communication, above alluded to, from Dr. Lempriere, who, in speaking of the disease in respect to Jamaica and St. Domingo, says, "Among the negroes and people of colour, it is of frequent occurrence." This he states in comparison to its infrequency among the white inhabitants, but which shews the disease to prevail much more than Dr. Buxton, from his inferences, is led to conclude. In fact, our information has been very defective on this subject in the hot countries, countries, the causes of which I shall not now enlarge upon, but conclude by a well authenticated anecdote.

A gentleman, high in the profession of physic, told me, about a fortnight ago, that, in conversation with a professional gentleman who had resided at Madeira many years, he was informed by the latter, that the disease of consumption was wholly unknown to the natives of that island. So heedless, it appears, are the best of us in regard to subjects that do not completely occupy our attention, or are not the immediate objects of our inquiry. In like manner, those authors to whom Dr. Buxton alludes, did not mention the disease of consumption, because they intended to give an account of the most prominent affections peculiar to those climates, of which consumption was not one, being a disorder of many climates, and about which they had taken no trouble to gain information.

Prepossession also goes a great way, and people in general do not seek for those things in a country which they have been previously told they will not find there. Thus, it has happened, that a general error has for a long time existed in regard to the prevalence of consumption in hot climates, because the opinion has been fixed, without sufficient investigation, that the disease either does not prevail there or is very infrequent.

Having now, with some certainty, established, that the hot climate is inimical to consumption, except in its incipient stage, I might take example by Dr. Buxton, and state the opposite temperature to be the most proper one for the treatment of the disease, as he has done, although in a contrary order. Such conclusion, however, would not quite accord with my experience and observation.

It may be considered to be a sort of paradox, that consumption should be so much detrimented in the same sort of climate, that has been found comparatively favourable to the cure of its incipient stage; especially as this disorder proceeds in a very regular course, varying only materially in its progress, by the augmentation of its symptoms, until the powers of life become exhausted and incapable of supporting the functions in which vitality consist. This I speak of the disease in this climate; in a hotter latitude, the symptoms in general became greatly more accelerated towards its termination. It may be said, indeed, that the heat of the climate increases the fever and colliquative sweats in the advanced stage; but, it ought also be equally considered, that it might operate to accelerate the more perfect formation of the symptoms in the incipient stage, which shew a disposition to take place early in the disease, as, in fact, every part part of consumption, in its ordinary occurrence, differs little from the others, excepting in the degree of intensity of the symptoms.

But, contrary to supposition, admitting the facts advanced, the disease in its early stage is considered to be capable of a more easy cure in a climate with these disadvantages, than with us: that is to say, whatever disadvantages may attend the hot climate to the consumptive in the early stage, something happens there to counterbalance and overcome them.

I must now beg to direct the reader's attention to this manifest difference in the situations of consumptive patients in this country and in such climates: that, in the latter, patients breathe the air of the atmosphere freely, without being subjected to the factitious process of encountering fire, or its being adulterated in close rooms, by undergoing frequent respiration without much change. Whereas, during a great part of the year, every thing in this country, in regard to air, in this disorder, is reversed. The common notion that all coughs must originate from cold, which last is most carefully to be avoided, when such disease takes place, of which Dr. Buxton has come forward as the champion, pervades the whole of this country, and becomes the foundation of every cure of cough. The exclusion of the atmosphere, in its most pure state, is therefore studiously prohibited, and its humidity diminished by fire, in order to arrive at a point of temperature considered merely to be salutary in opposition to all the supposed evils of cold.

One of the last statements that Dr. Buxton makes in his reply to me, and on which he appears much to rely, as favouring his practice of heat in consumption, is, by adducing proofs of the deaths of consumptive patients in various parts of the year. Thus, he finds more deaths in January and February to happen from this disease, than in the warmer weather. He, however, recollects, that these patients could not "generally" be attacked with the disease in the same winter and cold weather in which they die, because the disorder exists many months in its ordinary course before death takes place. If my readers think with me, that consumption lasts oftener six months, or thereabout, in this climate, than that it goes on ordinarily to a much more protracted period, the Doctor will find a difficulty in proving that the diseases of those who die in January and February (being the greatest number of deaths by this disorder of any, as I suppose, two months of the year,) commenced, and were chiefly caused by severe and variable weather, in his acceptation of the last epithet. He therefore must admit, that his chief cause of of consumption cannot have induced the commencement of those evils; and, even though we may take a period for the range of the disease protracted by some months, yet the conclusion is equally unfavourable to Dr. Buxton's hypothesis, in referring the commencement of consumption to the effects of severe and variable weather. In fact, the greater number of those who die in winter, must have been attacked with the disease in the warmer months; for, if we include March and part of April, it must be still more apparent, that the deaths in those months were caused by the disease which began in some part of the warm or hot months of the year.

It must be evident to those who may admit my ideas on the subject, why the deaths in consumption are not so frequent in the warmer months of the year in this country, because the consumptive have not arrived at the period when they must breathe an atmosphere much changed by fire and frequent respiration. As winter sets in, these evils are accumulated, often by necessity, and generally increased by the prevailing opinions of the benefits of warmth, and much augmented by those who may entertain Dr. Buxton's notions of the treatment of this disease. Hence the deaths became much more multiplied in winter.

I gave two cases of affection of the lungs, to shew the effects which might be expected from a lower temperature than has usually been thought beneficial. I did not intend there to shew the precise state of temperature I should judge to be the most convenient for the treatment of consumptive patients. I should certainly not recommend exactly the cold of a rigorous winter; but, in Dean's case, I judged that the degree of cold he would encounter, through the winter, would be very much less prejudicial to him, than the use of heat and an entire confinement to the house. Dean's case appeared to be that of impending consumption. The other case I adduced as an evident ulceration or abscess in the lungs, which got well in a much lower temperature than would generally have been recommended for this disease. Dr. Gladstone's case is also another example of a recovery from a state deemed to be consumption, in a very cold part of the year at Constantinople, for such it proved to be. Dr. Buxton appears to think, that the climate of that country must be mild in winter, in which particular he is much deceived, as, in consequence of the winds blowing at this time of the year over the vast tracts of land of Russia, the winter of Constantinople is full as severe as in England. Dr. Buxton has also employed himself in finding out what he conceives to be a geographical blunder, in my stating

Holland to be north of this country. This, however, would be of no consequence to the present discussion, as Holland is however to be colder; and there would have been little difficulty in understanding that I ranged Holland under the head of a country colder than England. But the truth of this important circumstance is, that I intended to copy, almost verbatim, from the Eclectic Review; without reflecting respecting geographical positions. Another apparent error, to which he also attaches some importance, has had its source from the same cause. Dr. Buxton also thinks he has discovered another inaccuracy of mine, in which he wishes to set me right, when I spoke of the temperature varying—evidently meaning by the expression to imply weather in which the temperature frequently changes. The Doctor conceives I mean the total variation of the thermometer, taking the whole of a certain proportion of time. I will not defend the accuracy of my expression, having stated what I meant by it. But this has led Dr. Buxton unsuspiciously to refute one of his own opinions. He finds the thermometer to vary, according to his acceptation of the term in England, in those months in which he thinks consumption prevails the least; and therefore infers, that the disease is not owing to that cause.

In accounting, however, for the greater frequency of consumption, as he supposes, in Jamaica, than in the other West-India islands, and in Sicily than in any other station in the Mediterranean, he states it to be his opinion, that this depends upon the greater variation of temperature in those islands, as indicated by the thermometer, in the ordinary way of reporting their observations.

I might swell out this paper to a very considerable length, if I were to notice all the illustrations of his opinions the Doctor has given in his last paper; this, however, I do not design to do, as I believe I have not passed by the most material of his remarks, in so far as they bear upon the question between us. For the same reason, I do not particularize every thing that I might say which may accord with my views, in regard to the treatment of this disease, especially as I have noticed some of these in my Letters on Consumption, and at the end of my Tract on Peritonitis.

I must not, however, conclude without saying a word on the sirocco wind, which, in my paper, was stated to be a dry wind. I have made enquiries on the subject from those who have been in the Mediterranean, and their accounts disagree in these particulars. But, whether very hot and dry or humid, it is not a fact on which much important conclusion depends. If I should be mistaken, I must beg to be excused, excused, by saying, that I have never been in the Mediterranean, by which I could be personally informed on the subject; and it is, for the same reason, that Dr. Buxton has so much mistaken the winter climate of Constantinople, he not having been there, if at all, as I suppose, in that season of the year.

THO. SUTTON.

*Crooms-Hill; June 10th*, 1815.

